# HMMSplicer: A Tool for Efficient and Sensitive Discovery of Known and Novel Splice Junctions in RNA-Seq Data

**DOI:** 10.1371/journal.pone.0013875

**Published:** 2010-11-08

**Authors:** Michelle T. Dimon, Katherine Sorber, Joseph L. DeRisi

**Affiliations:** 1 Department of Biochemistry and Biophysics, University of California San Francisco, San Francisco, California, United States of America; 2 Biological and Medical Informatics Program, University of California San Francisco, San Francisco, California, United States of America; 3 Howard Hughes Medical Institute, Bethesda, Maryland, United States of America; University of North Carolina at Charlotte, United States of America

## Abstract

**Background:**

High-throughput sequencing of an organism's transcriptome, or RNA-Seq, is a valuable and versatile new strategy for capturing snapshots of gene expression. However, transcriptome sequencing creates a new class of alignment problem: mapping short reads that span exon-exon junctions back to the reference genome, especially in the case where a splice junction is previously unknown.

**Methodology/Principal Findings:**

Here we introduce HMMSplicer, an accurate and efficient algorithm for discovering canonical and non-canonical splice junctions in short read datasets. HMMSplicer identifies more splice junctions than currently available algorithms when tested on publicly available *A. thaliana*, *P. falciparum*, *and H. sapiens* datasets without a reduction in specificity.

**Conclusions/Significance:**

HMMSplicer was found to perform especially well in compact genomes and on genes with low expression levels, alternative splice isoforms, or non-canonical splice junctions. Because HHMSplicer does not rely on pre-built gene models, the products of inexact splicing are also detected. For *H. sapiens*, we find 3.6% of 3′ splice sites and 1.4% of 5′ splice sites are inexact, typically differing by 3 bases in either direction. In addition, HMMSplicer provides a score for every predicted junction allowing the user to set a threshold to tune false positive rates depending on the needs of the experiment. HMMSplicer is implemented in Python. Code and documentation are freely available at http://derisilab.ucsf.edu/software/hmmsplicer.

## Introduction

RNA-Seq, which applies high-throughput sequencing technology to an organism's transcriptome, has revolutionized the study of RNA dynamics within a cell [1]. Millions of short read sequences allow both the presence and abundance of transcripts to be ascertained. RNA-Seq has been shown to have a better dynamic range for gene expression levels than microarrays [2] and enables scientists to view the transcriptome at single nucleotide resolution. Thus this technique combines the genome-wide scale of microarrays with the transcript variant detection power of Expressed Sequence Tags (ESTs).

RNA-Seq reads fall into two main classes: reads with full-length alignments to the genome and reads that span exon-exon junctions. Current sequencing runs produce tens of gigabases and it is likely that terabase sequences will be a reality in the near future. This massive output necessitates rapid techniques to analyze the data in a reasonable amount of time. For full-length alignments of sequence reads back to a reference genome, recent tools that rely on the Burrows-Wheeler Transform have yielded significant improvements in speed and accuracy. These include BWA [3], SOAPv2 [4] and Bowtie [5].

The more difficult RNA-Seq challenge is aligning reads that bridge exon-exon junctions since they by definition form gapped alignments to the genome with very short flanking sequence. These exon-exon junction reads reveal the exact location of splicing events, an intricate process wherein the intron in a pre-mRNA transcript is removed and the flanking exons are joined together. This tightly regulated process is coordinated by the spliceosome, a complex of many small-nuclear ribonuceloproteins (snRNPs) (reviewed in [6]). The spliceosome facilitates nucleophilic attack of the phosphodiester bond at the 5′ splice site (5′SS) by the branch point sequence. The 3′-hydroxyl at the 5′SS then reacts with the start of the next exon, the 3′ splice site (3′SS), ligating the exons and releasing the intron lariat. The branch point sequence, 5′SS and 3′SS are defined by short motifs within the intron sequence. In metazoans, the consensus splice site motifs are GTRAGT for the first six bp of the intron (5′SS) and YAG as the last 3 bp of the intron (3′SS). However, these motifs are extremely degenerate, leaving just ‘GT-AG’ as fairly reliable splice sites, found in 98% of known human introns [7]. Although most splicing in eukaryotic cells is performed by the spliceosome, non-spliceosomal splicing occurs and can be essential. One well-characterized example is the splicing of yeast *HAC1* and the homologous *XBP1* in metazoans [8]. In yeast, the transcription factor HAC1p regulates the unfolded protein response. HAC1p is, in turn, regulated by unconventional splicing of *HAC1* mRNA [9]. This splicing is not accomplished by the spliceosome. Instead, the protein Ire1p cleaves the *HAC1* mRNA in two places and the resulting edges are ligated with tRNA ligase [10]. In metazoans, *XBP1* is cleaved in a homologous manner, with the non-canonical splice boundaries CA-AG instead of GT-AG.

During the past decade, there has been a growing appreciation of the importance of alternative splicing as a mechanism for organisms to increase proteomic diversity and regulatory complexity (reviewed in [11] and [12]). The model of static exon and intron definitions yielding a single mRNA transcript and single protein sequence from each gene has proven overly simplistic. In reality, alternative splicing, the creation of multiple mRNA transcripts from a single pre-mRNA sequence by differential splicing, is extensive in multicellular organisms, increasing with organismal complexity. Recent RNA-Seq studies suggest that virtually all multi-exonic human transcripts have alternative isoforms [13], [14]. The extent of alternative splicing, as well as the balance between types of alternative splicing (*e.g.* alternate 5′SS versus exon-skipping splicing), differs by organism [15]. The regulation of splicing in different tissues and developmental stages, as well as the mechanisms for its regulation, is a subject of ongoing research [11], [16], [17]. Therefore, the ability to detect alternative splice isoforms with accuracy and sensitivity is key to comprehensive RNA-Seq analysis.

Aligning exon-spanning reads to the genome is difficult. Instead of a single full-length alignment, an algorithm must break a short read into two even shorter pieces and align each piece accurately. One early approach to short read splice junction detection was alignment using existing gene annotations, as done by ERANGE [18]. While this approach was necessary to align very short reads (36 nt or less) back to mammalian genomes, it does not address the question of novel junctions and cannot be used for organisms with incomplete or inaccurate genome annotations. Another early approach was to use BLAT [19], a tool developed for the alignment of longer EST sequence. This method can provide good results but requires extensive effort by the researcher to post-process and filter the search results, which could be achieved by the construction and training of a support vector machine specific to the organism and dataset [20]. In addition, BLAT searches on mammalian genomes can be slow.

The current leading algorithm for finding novel junctions in RNA-Seq data is TopHat [21]. TopHat uses full-length read alignments to build a set of exon ‘islands’, then searches for short reads that bridge these exon islands. The strength of this approach is that the resulting set of putative gene models can be used to estimate transcript abundance, as in the recently released Cufflinks software [22]. However, the algorithm must be able to define exon islands, which can be difficult when the coverage is low or uneven or when introns are small. While TopHat can find GT-AG, GC-AG, and AT-AC splice sites under ideal conditions, it does not extract any other splice sites. As a result, TopHat performs best on mammalian transcripts with relatively high abundance, but can stumble in more compact genomes and with non-canonical junctions.

Recently, several algorithms have been published that match reads more directly to the genome, including SplitSeek [23], SuperSplat [24], and SpliceMap [25]. SplitSeek divides the read into two non-overlapping anchors and initially detects junctions as places where the two anchors map to different places on a chromosome (i.e. the two exons with the intron between them), with no requirement for specific splice sites. These initial junctions are further supported by reads where only a single anchor maps to an exon - however, the requirement for at least one read split evenly across the exon-exon boundary reduces sensitivity in low coverage datasets and transcripts. Additionally, SplitSeek only supports ABI SOLiD reads currently. SuperSplat is another algorithm that reports non-canonical junctions (junctions with intron edges other than GT-AG, GC-AG, or AT-AC). However, this algorithm requires both pieces of a read to be exact matches to the reference sequence so it is not robust against sequencing errors or SNPs. SpliceMap divides reads in half, aligns each read half to the genome, then locates the remaining part of the read downstream within the maximum intron size. However, this algorithm considers only canonical splice junctions and requires read lengths of 50 nt or greater. In addition, although SplitSeek, SuperSplat, and SpliceMap all provide methods to filter the resulting junctions by the number and types of supporting reads, none provide a score that predicts the accuracy of a junction.

Here we introduce HMMSplicer, an accurate and efficient algorithm for finding canonical and non-canonical splice junctions in short-read datasets. The design of HMMSplicer was conceived to circumvent the inherent bias introduced by relying upon previously defined biological information. HMMSplicer begins by dividing each read in half, then seeding the read-halves against the genome and using a Hidden Markov Model to determine the exon boundary. The second piece of the read is then matched downstream. Both canonical and non-canonical junctions are reported. Finally, a score is assigned to each junction, dependent only on the strength of the alignment and the number and quality of bases supporting the splice junction. The scoring algorithm is highly accurate at distinguishing between true and false positives, aiding in novel splice junction discovery for both canonical and non-canonical junctions. HMMSplicer was benchmarked against TopHat and SpliceMap. It outperformed TopHat across a range of genome sizes, but most dramatically in compact genomes and in transcripts with low sequence read coverage. Compared to SpliceMap, it performed similarly in a human dataset and outperformed SpliceMap on an *A. thaliana* dataset.

## Results

### Algorithm Overview

**Figure 1:**
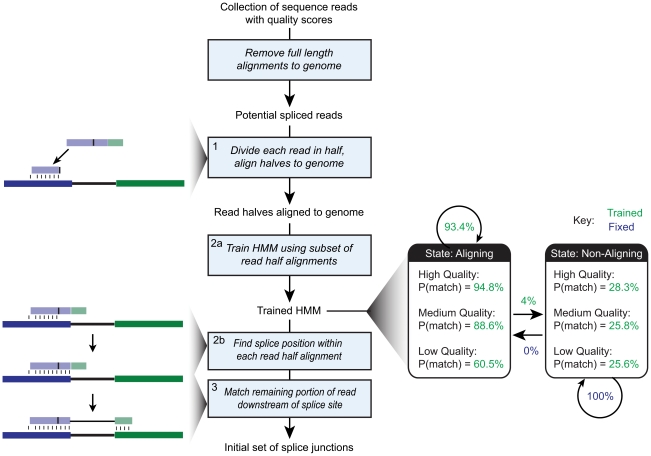
HMMSplicer pipeline. After removing reads that have full-length alignments to the genome, reads are divided in half and aligned to the genome (step 1 as defined in the Materials and Methods). The HMM is trained using a subset of the read-half alignments (step 2a). The HMM bins quality scores into five levels. Although only three levels are shown in this overview for simplification, the values for all five levels can be found in Table 1. The trained HMM is then used to determine the splice position within each read-half alignment (step 2b). The remaining second piece of the read is then matched downstream to find the other intron edge (step 3). The initial set of splice junctions then proceed to rescue (step 4) and filter and collapse (step 5) to generate the final set of splice junctions.

An overview of the HMMSplicer algorithm is shown in Figure 1. Before the HMMSplicer algorithm begins, full-length alignments to the genome are detected using Bowtie [5] and removed from the dataset. HMMSplicer begins by dividing the remaining reads in half and aligning each half to the genome. All alignments for both read halves are considered autonomously and are not resolved until the final scoring step. Once a read-half is aligned, a Hidden Markov Model (HMM) is used to detect the most probable splice position. The HMM is trained on a subset of read-half alignments to best reflect the quality and base composition of the dataset and genome. Next, the remaining portion of the read is aligned downstream of the exon-intron boundary, completing the junction definition. Finally, identical junctions are collapsed into a single junction and all junctions are scored, filtered by score, and divided by splice-site edges, with canonical (GT-AG and GC-AG) junctions in one result set and non-canonical edges in a second result set.

### Algorithm parameters

**Figure 2:**
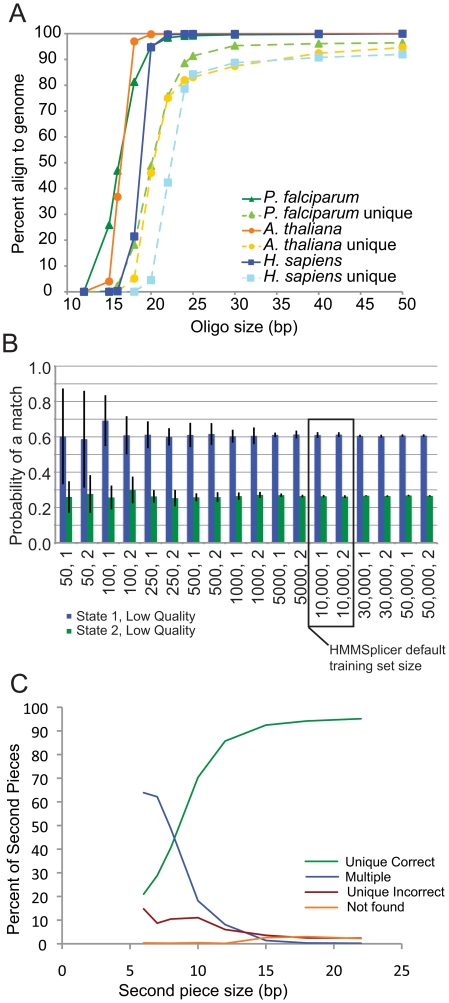
Algorithm parameters. **a**) Percent of oligos able to map within a genome as a function of oligo size. The solid lines show the percentages if oligos are able to map up to 50 times within the genome (the value used in HMMSplicer seeding). The dashed lines show the percentages if a unique match is required. **b**) HMM training. The values for the two most variable parameters of the HMM are shown here, with the x-axis representing different training set sizes and initial HMM parameters. The error bars show the standard deviation of ten repetitions of training. HMMSplicer uses a training subset size of 10,000. **c**) Effect of size, in bases, for the second piece of the read. The percent of second pieces uniquely mapping within 80 kbp of the first piece increases as the size of the second piece increases, while the percent of second pieces mapping to multiple locations decreases.

Our first step was to test the assumptions underlying HMMSplicer's algorithm by evaluating performance relative to key parameters: the required read length, the robustness of the HMM, and the ability to match the second piece of a read. First we examined the ability of read-halves to seed within a genome by measuring the fraction of read-halves aligned in the Bowtie read-half alignment step for various read-half sizes and genome sizes (Figure 2a). For the human genome, HMMSplicer performs optimally for reads 45 nt or longer (read-halves of 22 nt or longer), though shorter reads can be used. Simulation results, described below, confirm this assessment, showing a higher false positive rate when aligning 40 nt reads to the human genome. Next, we validated the robustness of the HMM training. An essential feature of HMMSplicer is that the HMM used to determine where the splice occurs within the read is trained from a subset of the input read set by an unsupervised algorithm. For the HMM training to be robust, it must train to similar values for an input read set, regardless of the initial values or the subset of reads used for training. This was validated using the human read set. Training sets ranging from 50 to 50,000 read-half alignments were used to train the HMM with two different sets of initial HMM values. For the first set of initial values, we used completely even values, i.e. a 50/50 probability of a match or mismatch for each quality score. For the second set, we used values close to those we expected as trained HMM values (Table 1). Training for each combination of training set size and initial value was repeated 10 times with different random subsets to measure the mean and standard deviation of the trained values. The results show that the HMM training converges on similar values regardless of training set size and initial values. The two most variable parameters are shown in Figure 2b, all other parameters showed less variability across the conditions (data not shown). Smaller training sets showed more variability so a default training set size of 10,000 was selected for HMMSplicer as sufficient to sample the space. Finally, mapping of sequences of various sizes within an 80 kbp maximum intron was analyzed to determine the optimal anchor size (Figure 2c). In the human genome, for sequences less than 8 nt in length, the most common result was multiple matches, whereas at 8 nt and above, a unique, correct match was the most likely result. Based on these data, the default anchor size was set at 8 nt for the default maximum intron size of 80 kbp. For compact genomes with smaller maximum intron sizes, such as the *P. falciparum* and *A. thaliana* datasets below, a shorter anchor size of 6 nt can be matched uniquely (data not shown).

**Table 1:** HMM Parameter Values.

	1→2	2→1	1: high	1: med-high	1: medium	1: med-low	1: low	2: high	2: med-high	2: medium	2: med-low	2: low
Initial Value Set 1	0.5	0	0.5	0.5	0.5	0.5	0.5	0.5	0.5	0.5	0.5	0.5
Initial Value Set 2	0.5	0	0.7	0.7	0.7	0.5	0.4	0.3	0.3	0.3	0.3	0.3
*A. thaliana* Trained Values	0.916	0	0.983	0.976	0.971	0.949	0.832	0.271	0.261	0.259	0.259	0.276
*P. falciparum* Trained Values	0.938	0	0.942	0.879	0.853	0.786	0.669	0.368	0.334	0.333	0.317	0.281
*H. sapiens* Trained Values	0.934	0	0.948	0.925	0.886	0.791	0.605	0.283	0.264	0.258	0.261	0.256

The initial and trained values for the HMM. The first two columns (“1→2” and “2→1”) show the probability of transitioning from State 1 to State 2 and the reverse. The probability of transitioning from State 2 to State 1 is fixed at 0 (indicating a 100% probability of remaining in State 2). For each state, the probability of a match at each quality bin is reported. The initial values were used to validate the HMM. HMMSplicer uses Initial Value Set 2, though the initial values do not impact the final trained values (see Figure 2b). The trained values are shown for each dataset analyzed. The Human values are the same as those shown in Figure 1, though in more detail.

### Benchmark Tests

HMMSplicer's performance was analyzed on simulated reads and three publicly available experimental datasets (Tables 2 and 3). The simulation dataset, generated from human chromosome 20, provides a measurement of the number of junctions detected and the false positive rate at different read lengths and coverage levels. However, simulation results do not model all the complexities found in experimental datasets, such as uneven coverage with a bias towards higher coverage of GC-rich regions, uneven distribution of sequence transversions, and inaccurate quality scores [26]. Three experimental datasets were selected from the NCBI Short Reads Archive (SRA), each representing a real world challenge. The first experimental dataset, ∼80 million reads from *Arabidopsis thaliana*, allowed analysis of HMMSplicer's performance using a dataset with low quality reads. The next experimental dataset, ∼14 million reads in *Plasmodium falciparum*, was used to assess performance in the context of uneven coverage and high AT content. The final experimental dataset, ∼10 million paired-end reads from *Homo sapiens*, was used to test HMMSplicer's performance in a larger metazoan genome. This dataset also provided a platform for analyzing transcripts with low abundance, alternative splicing and non-canonical splice sites.

**Table 2:** Simulation Results.

		HMMSplicer		TopHat	
Read Length	Coverage Level	# True Positives	% False Positives	# True Positives	% False Positives
**40 bp**	1	1484	0.7	451	1.1
	5	3478	1.3	1858	1.1
	10	3835	2.8	2825	1.2
	25	3908	4.7	3490	2.0
	50	3928	8.3	3630	3.3
**45 bp**	1	1630	0.2	503	0.2
	5	3634	0.8	2422	0.9
	10	3861	1.0	3458	1.3
	25	3928	2.2	3849	2.0
	50	3947	4.1	3901	3.8
**50 bp**	1	1701	0.2	457	0.7
	5	3646	0.3	2619	0.8
	10	3893	0.5	3579	1.1
	25	3943	1.1	3858	2.2
	50	3958	1.6	3908	3.1
**55 bp**	1	1711	0.3	390	0.8
	5	3677	0.5	2697	0.7
	10	3898	0.5	3581	1.1
	25	3948	1.1	3870	1.8
	50	3965	2.5	3915	3.1
**60 bp**	1	1684	0.1	433	0.9
	5	3671	0.3	2629	0.7
	10	3906	0.4	3581	0.8
	25	3951	0.9	3869	1.5
	50	3966	1.0	3930	2.9
**65 bp**	1	1698	0.1	405	0.7
	5	3684	0.4	2609	0.6
	10	3904	0.5	3525	0.8
	25	3945	1.0	3838	1.8
	50	3966	1.3	3928	2.4
**70 bp**	1	1629	0.1	1038	0.7
	5	3626	0.2	3297	1.6
	10	3893	0.5	3785	2.2
	25	3951	0.7	3931	6.5
	50	3960	1.2	3958	12.9
**75 bp**	1	1613	0.2	943	0.5
	5	3613	0.4	3101	0.5
	10	3899	0.5	3734	0.8
	25	3955	0.6	3939	1.5
	50	3966	1.2	3966	2.4

HMMSplicer and TopHat were run on read sets from 40 to 75 bp long at coverage levels from 1× to 50× on 503 non-overlapping gene transcripts from Human Chr20.

**Table 3:** Datasets.

	Accession Number	Number of Reads	Read Length	HMMSplicer time (min)	TopHat time (min)
***H. sapiens***	SRX011552(used for quality model)	N/A	75	N/A	N/A
***A. thaliana***	SRX002554	79,106,696	50	326	1162
***H. sapiens***	SRX011550	9,669,944paired end	45	880	645 ( or 271)
***P. falciparum***	SRX001454 SRX001455SRX001456 SRX001457	14,139,995	46*	108	188 (or 45)

*The 48-bp reads in the NCBI SRA set have a 2 bp initial barcode that was trimmed, resulting in 46 bp reads.

Datasets used for benchmark tests. For *H. sapiens* and *P. falciparum*, two times are given for TopHat. For *H. sapiens*, the longer time is with more sensitive settings, but the shorter time resulted in less than 5% fewer junctions at a similar specificity. For *P. falciparum*, the longer time is with more sensitive but less stringent settings whereas the shorter time is for the more stringent settings that resulted in significantly fewer junctions but with a much higher specificity.

### HMMSplicer combines high sensitivity with a low false positive rate

HMMSplicer was first tested on simulated read sets to determine its performance in an environment where true and false positive rates could be definitively measured. For the simulation, reads from 503 non-overlapping gene models on human chromosome 20 were generated at varying read lengths and coverage levels. For an accurate quality model, we used the error model from a human dataset [27]. In this read set, the second paired end read was extended to 75 bases, allowing us to simulate longer reads. The program maq was used to generate reads of length 40, 45, 50, 55, 60, 65, 70, and 75 bp at 1×, 5×, 10×, 25×, and 50× coverage [28]. TopHat was run on the same simulated dataset for comparison.

HMMSplicer's false positive rate was low overall, rising with short reads and high coverage (Table 2). The highest false positive rate, 8.3% was seen for 40 bp reads at 50× coverage, re-iterating the conclusion from parameter testing (above) that HMMSplicer performs ideally in the human genome with reads at least 45 bp long. At a length of 45 bp, the false positive rate for 50× coverage was 4.2%, while for reads 50 bp or longer the false positive rate never exceeded 2.5%, with most error rates remaining under 1%.

**Figure 3:**
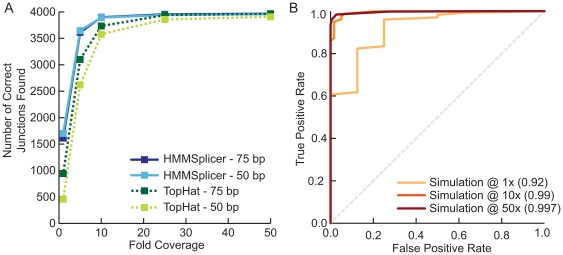
Simulation results. (**a**) Results for HMMSplicer and TopHat for 50 and 75 bp reads. Although values are similar at higher coverage levels, HMMSplicer exhibits substantial increases in sensitivity at lower coverage levels. (**b**) ROC curve for the 50 bp simulation results at 1×, 10×, and 50× coverage demonstrates that HMMSplicer's scoring algorithm accurately discriminates between true and false junctions. The number in parentheses is the area under the curve for each coverage level.

HMMSplicer was effective at identifying junctions, even at low coverage levels (Figure 3a). With 50 bp reads at 1× coverage, HMMSplicer was able to identify more than 40% of all the junctions in the set (1701 of 4043). At 5× coverage, more than 90% of the junctions were found (3646 of 4043). Higher coverage levels increase the number of junctions found, and at 50× coverage more than 98% of the junctions are found (3958 of 4043). While TopHat finds similar number of junctions at higher coverage levels, HMMSplicer finds three times as many junctions at 1× coverage with reads less than 70 bp long, and more than 50% more junctions with reads 70 or 75 bp long. Seventy-seven junctions were never detected by either program, even at 50× coverage and 75 bp reads. These junctions either had a homologous region within the genome or encompassed tiny initial or final exons that, because the simulated transcripts did not include UTR regions, had artificially low coverage.

One of HMMSplicer's strengths is that the algorithm provides scores for each junction, indicating the confidence of the prediction. To judge the accuracy of the scoring algorithm, Receiver Operator Characteristic (ROC) curves were generated comparing the true positive and false positive rate (Figure 3b). To measure true and false positive rate, simulation results for all scores were considered. Predicted junctions that aligned to the correct source of the simulated read were considered correct, while predicted junctions that aligned to another location were considered false. The ROC curves show that the HMMSplicer scoring algorithm was highly accurate, with the inflection point for 10× coverage and 50 bp reads including 98.7% of the true junctions and only 6.7% of the false junctions. At the default score threshold, 99.3% of true junctions and only 13.3% of the incorrect junctions were included.

### HMMSplicer performs well on datasets with low quality sequence reads

High-throughput sequencing datasets can have high error rates, however there is still useful data to be gleaned from these datasets. The first dataset, ∼79 million reads, each 50 bp long, in *Arabidopsis thaliana*, evaluated the performance of HMMSplicer with variable quality sequence reads [29]. *A. thaliana*, a model plant species, has a genome of 125 million base pairs with ∼25,500 protein-coding genes [30]. The mean exon and intron sizes are 78 bp and 268 bp, respectively, with an average of 4.5 introns per gene [31].

**Figure 4:**
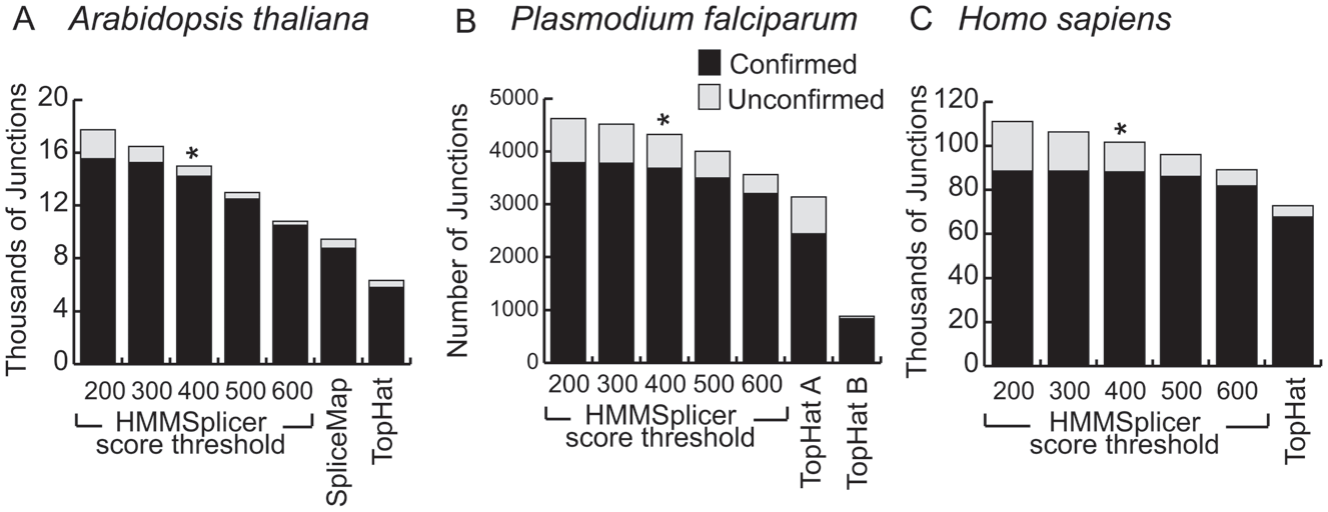
Overview of HMMSplicer and TopHat results in (a) *A. thaliana*, and (b) *P. falciparum* and (c) *H. sapiens*. For each dataset, HMMSplicer results are shown at five different score thresholds. The numbers on the bottom axis (200 to 600) are the thresholds for junctions with multiple reads; the threshold was set 200 points higher for junctions with a single read. The * indicates HMMSplicer's default score threshold. SpliceMap results are shown for the *A. thaliana* dataset only, as SpliceMap cannot be run datasets with reads less than 50 nt long. For *P. falciparum*, TopHat was run with two different parameter sets. TopHat A was run with a segment length of 23 resulting in more junctions but a lower specificity whereas TopHat B used the default segment length of 25 resulting in fewer junctions with more specificity.

We analyzed these low-quality reads, using a minimum intron length of 5 bp, a maximum intron length of 6 kbp, and an anchor size of 6 bp. The gene models in the most recent release of The Arabidopsis Information Resource (TAIR9, http://www.arabidopsis.org) contain introns from 3 bp to 11,603 bp long with 99.9% of the introns falling between 5 and 6,000 bp. At the default score threshold, HMMSplicer detected 14,982 junctions, with 95% (14,217) of the predicted junctions matching TAIR9 annotations (Figure 4a). The relatively low number of junctions found overall despite the size of the dataset is likely a result of low read quality. The low quality also decreases the HMMSplicer scores, causing a sharper decrease in the number of junctions at higher score thresholds compared to other datasets (Figure 4a).

TopHat and SpliceMap were also run on the *A. thaliana* dataset. TopHat, run with a minimum intron size of 5 bp and a maximum intron size of 6 kbp, was able to locate only 6,346 junctions, less than half the number found by HMMSplicer, with 91.7% (5,820) of these predictions matching TAIR9 annotations (Figure 4a). SpliceMap was run with the same 6 kbp maximum intron size (the minimum intron size is not configurable). SpliceMap found 9,438 junctions, 92.8% of which match TAIR9 annotations. Although SpliceMap found more junctions than TopHat, HMMSplicer found 50% more junctions than SpliceMap with a higher percentage matching TAIR9 annotations than either competitor.

### HMMSplicer performs well in datasets with uneven coverage

The *P. falciparum* genome is fairly compact and AT-rich, containing approximately 5,300 genes in 23 million base pairs [32]. In the latest genome annotation (PlasmoDB 6.3, http://www.plasmodb.org), the average exon size is 890 bp and the average intron size is 168 bp with an average of 1.54 introns per gene. Previous research on an earlier release of the genome annotation indicated that approximately 24% of the gene models predicted for *P. falciparum* are incorrect [33]. The malaria research community has focused on improving the genome annotation, and the most recent genome annotation release addresses many incorrect annotations. However, there are still numerous unconfirmed gene models with limited or no EST evidence.

The *P. falciparum* read set was published in the NCBI SRA following work on the Long March technique [34]. The dataset downloaded from NCBI SRA contains 14,139,995 reads, each 46 bp long. This dataset has uneven coverage with coverage varying significantly even within a single transcript. To detect splice junctions in this dataset, HMMSplicer was run with a minimum intron size of 10 bp, a maximum intron size of 1 kbp and an anchor size of 6 bp. This range includes 99.6% of the known introns in the current *P. falciparum* genome annotation. At the default score threshold, HMMSplicer identified 4,323 junctions in this dataset, 85.2% of which overlapped either known gene models or ESTs (Figure 4c). TopHat found 3,138 junctions in this dataset with 77.7% aligning to known gene models or ESTs. By re-running TopHat with more stringent alignment parameters, the percent of confirmed junctions was boosted to 94.8%, but this resulted in a 71% decrease in the number of found junctions (885). In contrast, the output of HMMSplicer can be filtered for more stringent confirmed junction percentages simply by raising the score threshold. SpliceMap could not be tested on this dataset because the reads are less than the minimum 50 nt length required by the algorithm.

### HMMSplicer performs well in large metazoan genomes

The *Homo sapiens* genome is large (3.2 billion base pairs with ∼25,000 genes), and contains both short exons (∼59 bp on average) and large introns (∼6,553 bp) [31], creating a significant challenge for identifying splice junctions. However, the human genome is well annotated with abundant EST evidence, allowing evaluation of HMMSplicer's performance on transcripts with low abundance, alternatively spliced junctions, and non-canonical junctions. Although the human genome is well studied, the complications of tissue-specific expression and widespread alternative splicing mean that many splicing events have not yet been detected. For our benchmark tests, we selected a human dataset containing 9,669,944 paired-end reads, each 45 bp long, from a single individual's resting CD4 cells [27]. The version of the genome used for analysis was the February 2009 human reference sequence (GRCh37) produced by the Genome Reference Consortium. Two reference sets were used to identify known introns. The first set represents known genes and well-studied alternates (genes present in the manually curated RefSeq [35]), while the second set represents a more extensive set of junctions, including many alternative splicing events (RefSeq genes and an additional 8,556,822 mRNAs and ESTs from GenBank [36]).

HMMSplicer was run with a minimum intron length of 5 bp and a maximum intron length of 80,000 bp, covering 99.1% of known introns in the human genome. Because HMMSplicer must match the second piece of the read downstream of the initial exon edge identified, the HMMSplicer algorithm is sensitive to maximum intron size. For efficient and accurate matching in 80 kbp introns, we used an anchor size of 8 nt, instead of the 6 nt anchor used in *A. thaliana*. At the default score threshold, HMMSplicer found 101,664 junctions, 87% of which (88,162) matched known genes or ESTs/mRNAs (Figure 4b). TopHat was run with the default intron size range of 70 to 500,000 bp, which covers 99.9% of known introns in the human genome. TopHat found 72,771 junctions, of which 93.0% (67,664 junctions) matched known genes or ESTs/mRNAs. Increasing the score threshold to 600 for junctions supported by multiple reads (800 for junctions supported by a single read) yields a similar confirmed junction rate of 91.8% and leads HMMSplicer to find 89,130 junctions, 22% more than TopHat.

Because this publicly available 45 nt dataset is too short for analysis by SpliceMap (which requires 50 nt reads), we were unable to directly compare HMMSplicer to SpliceMap on this dataset. Instead, we ran HMMSplicer on the human dataset analyzed in the SpliceMap publication [25], a set of 23,412,226 paired end reads of 50 nt each from a human brain sample (GEO Accession number GSE19166). SpliceMap is published as finding 175,401 splice junctions in this dataset with 82.96% EST validation. Filtering lowers the number of junctions found while raising the validation rate, so that at a validation rate of 94.5%, SpliceMap detected 121,718 junctions. HMMSplicer was run on the same dataset with default parameters, yielding similar results of 177,890 junctions with 84.2% EST validation at the default score threshold. Raising the score threshold to 800 (1000 for single junctions) we found 131,007 junctions with 94.5% EST validation. Our comparisons suggest that HMMSplicer finds slightly more (7%) junctions than SpliceMap at an equivalent EST validation level (94.5%) in this human dataset.

### HMMSplicer identifies many junctions in low abundance transcripts

A recent RNA-Seq study across 24 tissues in humans showed that ∼75% of mRNA in a cell is from ubiquitously expressed genes [37]. Furthermore, although transcripts from ∼11,000 to ∼15,000 genes were detected (depending on the tissue), the 1000 genes with the highest expression levels contributed more than half the mRNA in each tissue. The importance of RNA-Seq in the detection of novel splice junctions is not in these ubiquitous highly expressed genes, which generally have EST coverage, but in the tissue-specific genes with lower transcript abundance.

**Figure 5:**
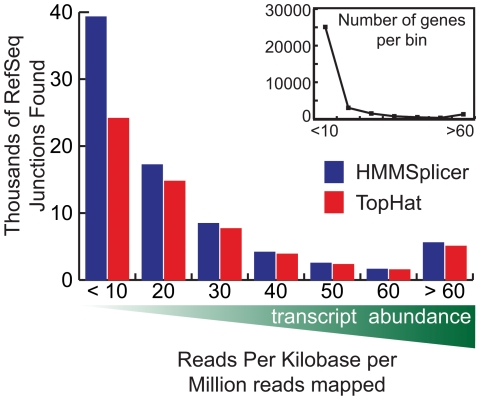
Human results compared by transcript abundance. Transcript abundance was measured as Reads Per Kilobase per Million reads mapped (RPKM) and the genes were binned by RPKM to show the number of RefSeq junctions found at different levels of transcript abundance. For genes with an RPKM less than 10, HMMSplicer found 76.2% more junctions, whereas for genes with an RPKM above 50, HMMSplicer found only 6.7% more junctions. While a smaller number of highly expressed genes dominate the mRNA population, 74.8% of genes have RPKM values less than 10.

Therefore, we measured HMMSplicer's capacity for detection of junctions in low-abundance transcripts in the human resting CD4 cell dataset. In RNA-Seq experiments with non-normalized cDNA samples, the coverage level of a gene varies depending on relative transcript abundance. A convenient measure of read coverage relative to the transcript abundance is Read Per Kilobase per Million reads mapped (RPKM) [18] which counts the number of reads that map to a gene, normalized by the length of the gene in kilobases, per million reads mapped to the genome. Figure 5 shows the number of predicted junctions matching RefSeq-defined introns at different RPKM levels. HMMSplicer identified more junctions than TopHat at all RPKM levels, but the difference is greatest at low values of RPKM. This is relevant to many RNA-Seq experiments. In this dataset, 75% of genes had an RPKM of 10 or less.

### Sequence-level analysis reveals alternate 5′ and 3′ splice sites

**Figure 6:**
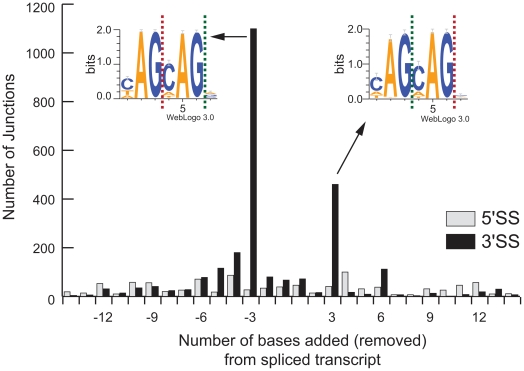
Alternative 5′ and 3′ splice sites. HMMSplicer results within 15 bp of RefSeq introns were analyzed to measure the number of bases added or removed from the spliced transcript. There were 997 instances where the intron had an alternate 5′ splice site (5′SS, shown in grey) and 2,577 instances of an alternate 3′ splice site (3′SS site, shown in black). The most common alternative splice was 3 bases removed or added to the exon at the 3′SS. TopHat results showed a similar pattern, though only 875 alternates (262 5′SS alternates and 613 3′SS alternates) are found, less than a quarter of the HMMSplicer results. WebLogos were constructed from the sequences at the 1,099 alternate 3′SS with three bases removed from the transcript and the 460 alternate 3′SS with three bases added to the transcript. For these, the green dashed line shows the alternate splice site while the red dashed line shows the canonical splice site. In both cases, a repetition of the YAG splice motif is evident.

HMMSplicer's approach allows discrimination of closely spaced alternative splice sites, providing a method to study fundamental questions about the biology of splicing which have not yet been addressed with RNA-Seq experiments. Alternative splicing analysis in RNA-Seq data frequently focuses on quantifying isoform expression level, such as in a recent study measuring isoform abundance based on relative coverage levels of exons [38]. This is an important application, but the sequence-level detail of RNA-Seq data provides the power to examine alternative splicing at a finer level of detail. Analysis within the human resting CD4 cell dataset showed instances where splice sites varied slightly from known intron boundaries, suggesting an inexact splicing event. To investigate these results further, all junctions overlapping RefSeq introns with fewer than 15 bp differences in splice sites were examined and the number of bases added or removed from the exon boundary was counted. Overall, there were 997 instances (1.4% of junctions which match RefSeq) where an intron possessed an alternate 5′SS and 2,577 (3.6% of junctions which match RefSeq) instances of an alternate 3′SS. Alternative splicing which maintained the reading frame (*i.e.* added or removed a multiple of 3 bases from the transcript) was clearly preferred for the 3′ splice site (Figure 6). This result is not surprising given that the 3′SS motif, YAG, is shorter and shows more variation than the 5′SS motif, GTRAGT [6]. To investigate this result further, WebLogos [39] were constructed from the sequences at the alternate 3′SS that were off by 3 bases. Analysis of these WebLogos found at the alternate 3′SS shows repetition of the splice motif (*i.e.* YAGYAG).

### HMMSplicer identifies non-canonical junctions

We next analyzed the ability of HMMSplicer to identify junctions with splice sites other than GT-AG using the human resting CD4 dataset for analysis. The most common splice sites, GT-AG, GC-AG, and AT-AC, are found in 98.3%, 1.5% and 0.2% of human introns, respectively [7]. By default, HMMSplicer attempts to adjust intron edges to GT-AG, GC-AG or AT-AC but includes only GT-AG and GC-AG introns in the set of canonical junction predictions. The user can alter the splice sites for adjustment and filtering or can eliminate these steps entirely. We examined the splice sites in junctions found by HMMSplicer. Counting only junctions that matched known mRNA/ESTs, HMMSplicer detected 87,245 GT-AG junctions, 791 GC-AG junctions, and 97 AT-AC junctions. This is 99% GT-AG, 0.9% GC-AG, and 0.1% AT-AC, which corresponds well with the published rates. The ratio of junctions that match known junctions is much lower for non-GT-AG junctions (20.3% for GC-AG and 6.5% for AT-AC). To resolve whether HMMSplicer non-canonical junctions are false positives or novel instances, further experimental validation will be required. Regardless, HMMSplicer provides all junctions and allows the user to filter based on the experiment's objectives.

**Figure 7:**
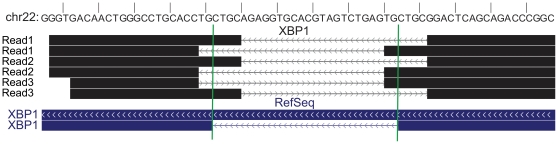
XBP1 non-canonical intron. HMMSplicer discovers the non-canonical *XBP1* intron. HMMSplicer identifies three reads containing the non-canonical CA-AG splice site in *XBP1*. Because the reads are fairly evenly split, both read-halves aligned to the genome. The edges identified by HMMSplicer are 2 and 4 bp off from the actual splice site because the sequence at the beginning of the intron repeats the sequence at the beginning of the subsequent exon. When identical junctions are collapsed, there are two junctions, one with a score of 1024 and one with a score of 1030, which puts them in the top 0.5% of the collapsed non-canonical junctions.

Although rare, there are also splice junctions that do not have GT-AG, GC-AG or AT-AC splice sites. For example, the *HAC1* mRNA and its metazoan homologue *XBP1* are spliced by Ire1p with the non-canonical splice sites CA-AG, initiating the unfolded protein response [8]. HMMSplicer's non-canonical junction results on the human dataset contained three reads spanning the *XBP1* non-canonical intron with scores ranging from 927 to 971 (Figure 7). The sequence at the beginning of the intron is identical to the initial exon sequence, so the HMM was unable to resolve the exact junction edges correctly. This resulted in two possible predictions, one 2 bp upstream from the actual site and one 4 bp downstream from the actual site. Collapsing identical junctions resulted therefore in two junctions, one with a score of 1024 and one with a score of 1030, which put them in the top 0.5% of the collapsed non-canonical junctions.

### HMMSplicer finds true novel junctions in genomes with incomplete annotation

**Figure 8:**
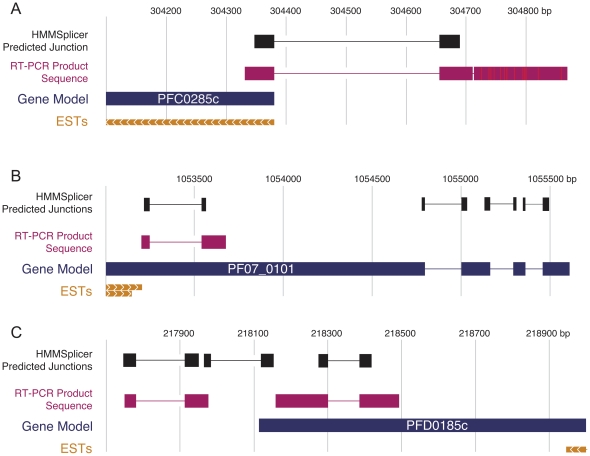
Experimental confirmation of predicted *Plasmodium falciparum* splice junctions. Schematics of the predicted splice junctions and sequenced RT-PCR products for **a**) PFC0285c, **b**) PF07_0101, and **c**) PFD0185c. For PFC0285c, the verified junction likely splices an additional exon in the 5′UTR to the coding region of the gene. The confirmed junction in PF07_0101 splices out 291 nt (97 aa) from the first exon, which could represent an alternative protein-coding isoform, or an error in the gene model. The demonstrated junctions in PFD0185c excise 85bp near the 3′ end of the gene, causing a frameshift, and appear to splice two exons within the 3′UTR of the gene together. Again, the junction within the gene model may represent an alternative splicing event or an error in the gene model. ESTs near all three areas are included to provide the direction of the genes.

To determine if unconfirmed junctions predicted by HMMSplicer represent true novel junctions or false positive predictions, we experimentally validated four previously unknown junctions predicted from the organism with the least thorough annotation, *P. falciparum* (Figure 8). All four junctions were relatively high scoring but no EST or experimental data exists for comparison, and each case conflicts with the current PlasmoDB gene model. The first junction (score = 1300), in PFC0285c (predicted to encode the beta subunit of the class II chaperonin tailless complex polypeptide 1 ring complex), suggests an additional exon at the 5′ end of the gene model, possibly belonging to the 5′ untranslated region (UTR). The second junction (score = 1198) belongs to PF07_0101, a conserved *Plasmodium* protein of unknown function. This previously unknown junction excises 291bp out of the middle of the first annotated exon, which would result in a protein 97 amino acids (aa) shorter. The third and fourth junctions, with scores of 1261 and 1175, respectively, are in PFD0185c, another gene of unknown function conserved across *Plasmodium* species. One junction lies within the predicted gene, splicing out 85bp and leading to a frameshift near the 3′ end, while the other appears to splice together two exons in the 3′UTR. RT-PCR followed by sequence analysis verified all four splice junctions predicted by HMMSplicer (Figure 8), confirming HMMSplicer's ability to predict true novel junctions from RNA-Seq data.

## Discussion

HMMSplicer is an efficient and accurate algorithm for finding canonical and non-canonical splice junctions in short read data. Our benchmark tests on simulated data and three publicly available datasets show that HMMSplicer is able to detect junctions in compact and mammalian genomes with high specificity and sensitivity. The real world challenges in these datasets include low quality reads and uneven coverage. Built on Bowtie, HMMSplicer is fast, comparable in CPU time to TopHat. Analysis also demonstrates HMMSplicer's ability to find splice junctions on transcripts with low abundance, alternative splicing, and non-canonical junctions.

Comparisons with TopHat show that HMMSplicer is able to find more junctions with a similar level of specificity in each of these datasets. Comparisons with SpliceMap show that HMMSplicer has similar performance, yielding slightly more (7%) EST matching junctions in paired-end human datasets. However, in the low sequence quality *A. thaliana* dataset, HMMSplicer significantly outperforms SpliceMap. HMMSplicer was not compared to SplitSeek [23] as this algorithm only processes colorspace reads. Though the algorithm is similar, we anticipate that HMMSplicer would be more sensitive than SplitSeek, since this algorithm requires at least one read to be split evenly across the splice junction. HMMSplicer, TopHat, and SpliceMap are all free from this constraint. Finally, the SuperSplat [24] algorithm is the only other currently available algorithm that detects non-canonical junctions to our knowledge. Unfortunately, the current version of SuperSplat does not align reads with any mismatches, and also has large memory requirements (5–32 GB to index the *A. thaliana* genome).

A major strength of HMMSplicer is that it is the only software package that provides a score for each junction, reflecting the strength of the junction prediction, which allows tuning of HMMSplicer's results to an experiment. While many splice junction algorithms filter on specific attributes to improve validation rates, for example, SpliceMap has filtering to remove junctions with only a single supporting read, HMMSplicer's score provides a more flexible way to tune true and false positive rates for the experiment. The score is based solely on the number of bases on each side of the junction, the quality of those bases, and the junction's similarity to potential full-length matches. Re-annotation experiments would necessitate a higher threshold to avoid false positives, but experiments looking for novel junctions could use a lower threshold to include as many true positives as possible. The threshold can also be tuned for non-ideal datasets, such as the low quality *A. thaliana* dataset. The score is highly predictive despite the fact that it does not include biological factors such as splice site or intron length in its calculation, making it ideal for detection of novel splice junctions.

Alternative splicing is an area of intense research where HMMSplicer's approach provides a significant advantage over algorithms that rely on exon islands, such as TopHat. In the case of alternate 5′ or 3′ splice sites, the major isoform may mask the signal from a minor isoform, especially in genes without high sequence coverage. HMMSplicer accurately identifies small variations in 5′ and 3′ splice sites. These small variations in splice sites, most frequently 3 nucleotides added or removed from the transcript at the 3′ splice site (1 amino acid added/removed from the translated protein), demonstrate how the repetition of the splice motif can cause inexact splicing. HMMSplicer's unbiased approach to alignment, combined with the sequence level power of RNA-Seq, has enormous potential for biological inquiry into alternative splicing.

The depth of RNA-Seq and the unbiased approach of HMMSplicer also allow investigation into non-canonical splicing. HMMSplicer allows the researcher to define canonical splice sites, and returns both canonical and non-canonical results. Scores in HMMSplicer's predicted junctions aid the discovery process, as evidenced by the *XBP1* example in the human dataset. In HMMSplicer's results, it was ranked in the top 0.5% of the non-canonical splice results.

In conclusion, HMMSplicer is a valuable addition to the algorithms available for finding splice junctions in RNA-Seq data. The software, documentation and details about the datasets and analysis can be found at http://derisilab.ucsf.edu/software/hmmsplicer.

## Materials and Methods

### Algorithm Description

The HMMSplicer algorithm has four main steps: seeding reads within the reference genome, finding the splice position, matching the second piece of the read, and scoring/filtering splice junctions. Figure 1 shows an overview diagram of the HMMSplicer pipeline.

As a pre-analysis step, dataset reads are aligned to the reference genome using Bowtie [5]. Reads with full-length alignments to the genome contain no junctions and are therefore removed from consideration. These genome-matching reads may be used to build a coverage track that can be viewed in the UCSC Genome Browser [40] or other applications.

#### Step 1. Read-half alignment

To determine the read's seed location within the genome, we assume that each read spans at most a single exon-exon junction. Reads are divided in half, rounding down for reads of odd length, and both read-halves are aligned to the genome using Bowtie (current version 0.12.2), although other full-length alignment algorithms may also be used. This approach will locate an alignment for both read halves if the read is somewhat evenly split across a junction, and these alignments are carried through the algorithm independently until they are resolved during scoring. However, if the read matches unevenly across the junction (*e.g.* if one side of a 45 nt read is 35 nt long and the other side is 10 nt long, referred to as a “35/10 split”), only the longer side will be seeded in this step. A read-half may not align if the larger half falls on another exon-exon junction or if sequencing errors prevent an alignment. Alternatively, a read half may have multiple alignments. As long as the duplicates are below a repeat threshold (50 alignments by default), all seeds are continued through until the filtering part of the algorithm; duplicate junction locations for a read are resolved at that point. For clarity in the text below, the half of the read that seeded will be referred to as the ‘first half’ and will be described as if the initial half of the read matched to the 5′ edge of the intron, with all sequences in the sense direction. In reality, either half of the read could match to either edge of the intron.

#### Step 2. Determine Splice Site Position

The alignment of a read-half determines an outside edge of the spliced read alignment, but does not determine where the exon-intron boundary occurs. To return to our previous example read with a 35/10 split, the first half of the read, corresponding to 22 bases, will be aligned but it will be unclear that the first side extends to 35 bases. A simplistic approach to this problem would be to extend the seed until a mismatch occurs but this approach ignores both the additional information available in quality scores and the high error rate inherent in many high-throughput sequencing technologies. Continuing from the 35/10 split example, imagine, after the first 22 bases of read-half, there is one mismatch to the genome at a low quality base and then 12 bases in a row which match the genome. The simplistic approach would be to assume the read stopped aligned after the first mismatch, suggesting the split is 22/23 instead of 35/10, resulting in an incorrect junction alignment. To avoid this type of error, HMMSplicer utilizes a two-state Hidden Markov Model (HMM) to determine the optimal splice position within each read. State 1 describes a read aligning to the genome. In this state, we expect that most bases in the read match their partner in the genome, and that the probability of matching will vary based on the read base quality (high quality bases are less likely to be sequencing errors and thus more likely to match). State 2 is cessation of alignment to the genome. In this state, matches between the read and the genome are essentially random and do not depend on quality. For example, a genome with a GC content of 50% would yield an expected probability of 25% for each base to match the target genome location, regardless of sequence quality score. The most probable transition point from State 1 to State 2 defines the optimal splice position. In the 35/10 split example, the HMM would evaluate the probability of a 22/23 split, with 10 matches in a row in State 2 (where the probability of a match is only 25%) compared to the probability of a 35/10 split where a low quality base causes a single mismatch while remaining in State 1. Assuming the probability of a mismatch in State 1 in a low quality base was about 30% (a typical value), the 35/10 split would be more probable than the 22/23 split. (All other possible splits would also be considered, but these would be low probability compared to the 22/23 and 35/10 split options.)

Within each state of the HMM, the quality is binned into five levels, representing low, medium-low, medium, medium-high, and high quality scores. Using five bins provides the best balance between having sufficient bins to distinguish quality levels, while maintaining enough bases within each quality bin that the HMM can be adequately trained using a random subset of reads. Using a separate bin for each quality score created situations where one or more quality score were under-trained because quality scores are not evenly distributed from zero to forty. Increasing the training subset size can ameliorate this problem, however results with more quality bins were not significantly better than results with five quality bins (data not shown).

The HMM is trained on a randomly selected subset of the input read set. The training is accomplished using the Baum-Welch algorithm [41], an expectation maximization technique that finds the most likely parameters for an HMM given a training set of emissions. For HMMSplicer, emissions are strings of match/mismatch values derived from the alignment of the whole read to the genome at the position of each seed match. By using an unsupervised training method, the HMM values can be trained without additional input from the user, such as known genome annotations. This allows for a more sophisticated approach than the simplistic model described above while maintaining model unbiased by additional information such as known genome annotation. This training allows the values to be optimal for any particular genome and sequencing run. For example, genome specific training can adjust for biases in genomic nucleotide composition. One of the datasets used in our testing is *P. falciparum*, which has a genome that is 80% AT. This reduced complexity makes the probability of a match in random sequence higher than the 25% that it would be in a genome with balanced nucleotide distributions. In addition, training provides a way to validate the model. The premise behind the model is that in State 1 the probability of a match should increase with the quality of a base, but in State 2 the probability of a match should be independent of the quality score. If this model is accurate then regardless of initial values, the trained HMM should reflect this expectation. The outcome of the training, detailed in the Results section above, confirm the robustness of the model to different initial values. The HMM values for each parameter, before training and after training with each dataset studied, are given in Table 1. For each organism, the model trains as expected. Parameters in State 1 show a higher rate of matches than mismatches, varying by quality score, while parameters in State 2 remain at approximately 25% probability of a match regardless of quality. The only exception is for *P. falciparum*, where the probability of a mismatch in State 2 varies from 37% to 28% depending on quality because of the 80% AT bias in the genome.

After the HMM is trained, it is run for every read-half alignment, yielding the coordinates of the first piece of the read alignment, including the first exon-intron boundary of the splice junction. In the event of multiple equally probable splice positions, the splice position with the shortest second piece is selected. A falsely short second piece may still match within the maximum intron distance and has the potential to be adjusted to the correct splice site in the canonical splice-site adjustment (see below for details). On the other hand, a second piece with false bases added to the beginning will likely not match within the maximum intron distance causing the read to be discarded. If the remaining part of the read is too short (eight nucleotides or fewer by default), the alignment is set aside. Uncertainty in the precise location of the splice junction and short alignment can be further resolved in a subsequent evaluation process described below.

#### Step 3. Determine Spliced Exon position

Once the splice position has been determined, the first exon-intron boundary has been identified. To determine the second exon-intron boundary, the remaining part of the read, (the ‘second piece’), must be aligned. To reduce search space to a manageable and biologically relevant size, a default of 80 kbp downstream of the initial alignment is considered, although the user may adjust this to the most appropriate value for the organism and experiment. HMMSplicer first determines potential location positions by using the initial eight nucleotides of the second piece as an anchor (this anchor size may also be tuned to the organism and experiment), searching for all locations within the maximum intron size where this anchor matches exactly. To accommodate possible sequencing errors in these initial eight nucleotides, exact matches for the next eight nucleotides (*i.e.* positions 9–16 of the second piece) are found and are added to the set of anchors. For each position where an anchor has an exact match, the entire second piece of the read is compared to the genome and the number of mismatches is counted. The alignment with the fewest mismatches is selected as the best match. In the event of multiple best matches, the read is set aside to be resolved later.

At this point, a preliminary splice junction has been defined. However, the exact splice positions may be offset from the actual intron-exon boundaries by a few nucleotides, especially in cases where the sequence at the beginning of the intron matches that at the beginning of the second exon. In these cases, sequence alone cannot define the correct edges. To aid in correct splice edge definition, HMMSplicer uses an assumption about the biology of splice sites. The most common splice sites, GT-AG, GC-AG, and AT-AC, are found in 98.3%, 1.5% and 0.2% of human introns, respectively [7]. By default, HMMSplicer uses these three splice sites (in order of their frequency of usage) to adjust intron-exon boundaries, though the sequences can be changed or the feature can be turned off entirely. Given the frequency of these three splice sites compared to other splice sites, the use of splice sites for intron-exon boundary adjustment introduces a conservative assumption and can help resolve small ambiguities in the position of the splice site prediction. To perform the adjustment, both splice edges are moved an equivalent number of nucleotides to reach a canonical splice site, where possible. Junctions already at canonical edges and junctions that cannot be adjusted to canonical edges remain unchanged.

HMMSplicer provides a score for each predicted junction that does not rely on any biological information or assumptions about splicing machinery beyond the user-configurable adjustment to canonical splice sites, leaving the user free to apply the appropriate data processing filters for the experiment. The goal of the scoring approach is to use available information maximally while minimizing assumptions. For example, a score that incorporated the intron size distribution of the organism could have been more accurate, but would have introduced a strong bias toward typical intron sizes. Similarly, a scoring algorithm that penalizes non-canonical junction edges would have introduced a bias towards canonical splice sites. Instead, HMMSplicer's score uses information only about the genome sequence, read sequence, read quality, and splice position to derive a score. The researcher can introduce further filtering to the result set, based on the needs of the experiment, but the score is free from these biases.

To accomplish this goal, we chose an information-based approach to the score algorithm, akin to a BLAST bit score rather than the probability-based E-value [39]. The initial step of the scoring algorithm is to measure the amount of information in the alignment of one side of the junction read. Assuming each possible nucleotide is equally likely and the reported read nucleotide was certain (no sequencing errors) there would be four equally possible nucleotides at each position of the read, resulting in 2 bits per position (log_2_(4)). However, the reported nucleotide is not certain, and this uncertainty is encoded by the quality of the nucleotide. To scale for this, we multiply the 2 bits by the probability that the nucleotide call is correct, given the quality score. The sum of the information in each matching position of the read piece alignment is then used as the score for that read piece.

Given:
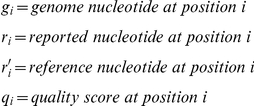



Score for one side is calculated as:
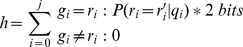
(1)


Both sides of the junction are scored using equation (1). To combine the scores for the individual read pieces, they are multiplied, giving a strong bias to evenly split reads. The score increase is greater for evenly split reads than for reads with uneven piece sizes. For example, comparing 50 nt reads and 70 nt reads, a 10/40 split compared to a 10/60 split will, under ideal conditions, raise the score from 400 to 600. By contrast, a 25/25 split compared to a 35/35 split will raise the score from 625 to 1225, a much more dramatic increase. This increase reflects the fact that a 10/40 to 10/60 split does not increase the information available as much as a 25/25 split to a 35/35 split.

Next, the score is corrected for the similarity to a full-length alignment. For each junction, if we hypothesize that the junction may actually be a full-length alignment, there are two possible positions for this alignment, either the left side is correct and the right side should be moved left adjacent to it, or the reverse. Both these possible full-length alignments are scored and the better alignment is kept. Half of this score is subtracted from the initial junction information as follows:
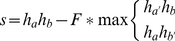
(2)


Where h_a_ is the score for the left side, h_b_ is the score for the right side, h_a′_ is the score for the left side when moved adjacent to the right side and h_b′_ is the score for the right side when moved adjacent to the left side. F is set to 0.5, an empirically derived value that gives the best score results when tested on the human dataset (data not shown).

As a final step, the scores are normalized to the range 0–1200, with most scores less than 1000 in practice. This is simply for easy visualization in the UCSC Genome Browser. The BED file output from HMMSplicer can be uploaded directly to the UCSC Genome Browser, which uses grey-scale to represent scores from 0–1000. To perform this scaling, the multiplier is 1200 divided by the theoretical maximum score for a read of the given length. When calculating the theoretical maximum, equation (1) reduces to the length of the read piece times 2 bits. Thus, if the read length is even, the multiplier is:
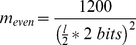
(3a)If the read length is odd, the multiplier is:
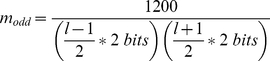
(3b)


All together, the full equation for the score value is:
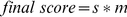
(4)


Once splice junctions have been detected and scored, HMMSplicer resolves instances where both halves of a read were aligned independently, as well as instances where one or both read halves created multiple alignments. For reads where independent read half alignments converged on the same junction position, a single copy of the junction is saved. For reads where the read halves had multiple seed positions, if one position has a score much higher than the other(s), that position is retained. If a read matches in multiple positions and all positions have close scores (by default, scores with differences less than 20, but this is user configurable), reads are saved in a separate set of output results reserved for duplicates.

#### Step 4: Rescue

Reads that cannot be matched uniquely can be used to lend support to a junction previously identified in the dataset. HMMSplicer attempts to rescue matches where the location of the first piece of the read is uniquely identified, but the location of the second piece is not. There are two sources of such reads: 1) reads with a second piece fewer than eight bases long and 2) reads where the second piece matched equally well to multiple locations within the maximum intron size. In both cases, HMMSplicer can apply the information from mapping the initial part of the read to rescue the read using other junctions found in the dataset. If another read ends at the same point as this read (i.e. has the same junction edge on the known side), the algorithm examines the other side of the junction to determine if the initial bases of the exon sequence match the second piece of this read. If so, this junction is assumed to be the source of the read.

#### Step 5: Filter and Collapse

Finally, initial junction-spanning reads are filtered and collapsed to yield a final set of predicted junctions. Splice junctions are divided into populations that do and do not match the most frequent splice sites (‘GT-AG’ and ‘GC-AG’ by default). Regardless of whether the user chooses to impose these splice site position sequences into the search, nonconforming junctions are saved and ranked separately. All reads creating the same intron are collapsed into a single junction with the score for these reads increased in relation to number of additionally covered bases. Distinct reads covering the same junction add significantly to a its potential to be real, but two identical reads may be from the same source, such as PCR amplification artifacts. To follow the previous example, a 35/10 split (35 bp on the first exon, 10 bp on the second exon) combined with another 35/10 split would not increase the score, but the 35/10 split plus a 10/35 split would yield a substantial boost to the score because the covered bases would now be now 35/35. To be exact, imagine the 10/35 junction read has a score of 800 and the 35/10 junction read has a score of 600. The higher score read is considered first, then the second read is collapsed onto it. In this case, the new junction adds 25 bases out of a total of, now, 70 bases covered, so a value of (25 / 70) * 600 is added to the original score of 800, yielding a collapsed score of 1214.2.

Collapsed junction predictions are then filtered by score. Multiple error-free reads spanning the same splice junction align to the correct splice site, facilitating determination of splice boundaries. In contrast, because sequencing errors are distributed throughout the read with three possible wrong base substitutions, reads with errors that create false positive junctions tend to be scattered as single, incorrect alignments. Previous studies concur that true junctions are more likely than false junctions to be covered by more than one read [42]. Therefore, junctions covered by a single read are evaluated more stringently than junctions covered by multiple reads, with a higher score threshold set for junctions covered by a single read. The default score thresholds for HMMSplicer are 600 for junctions covered by a single read and 400 for junctions covered by multiple reads. These score thresholds were optimal for the benchmark datasets, but ultimately the score threshold will depend on the number of reads used in the experiment (datasets with more reads may require higher score thresholds) and the purpose of the experiment (re-annotation studies will require higher score thresholds than studies looking for novel junctions).

### Benchmark Methods

For the benchmark tests, all analysis was performed on an 8-core Mac Pro with 16 GB of RAM. HMMSplicer was run with default parameters unless otherwise noted. TopHat version 1.0.12 was used. TopHat was run with the best parameters for the dataset/organism, though the only parameter found to have a large effect on results was segment length. For reads shorter than 50 nt, segment lengths of half the read length were used for TopHat, as it was found to dramatically increase the number of splice junctions found (i.e. 30,381 junctions identified for the default segment length of 25 versus 68,946 junctions identified with a reduced segment length of 22 in the human dataset). For the simulation dataset, TopHat was run with the default parameters, except with a segment length of 20 and 22 for reads 40 and 44 nt long. The *A. thaliana* dataset was run with default parameters except for a minimum intron size of 5 and a maximum intron size of 6000. The *H. sapiens* dataset was run with a segment length of 22 using the butterfly search and microexon search parameters. The *H. sapiens* dataset is paired end and, based on information in the publication [27], an inner mate distance of 210 was used. SpliceMap was run on the *A. thaliana* dataset by the SpliceMap first author using a 6 kbp maximum intron size (personal communication).

For most of the analysis, canonical splice junction results from HMMSplicer were used (*i.e.* GT-AG and GC-AG splice sites), as they are most comparable to results from other algorithms. Table 3 contains general characteristics of the datasets downloaded from NCBI SRA, including accession numbers.

### Experimental Validation in *Plasmodium falciparum*

#### Cell culture, RNA preparation, and poly-A selection

*Plasmodium falciparum* 3D7 Oxford parasites were sorbitol synchronized in early ring stage, then synchronized again 24 and 32 hours later for a total of 3 synchronizations during 2 consecutive cell cycles. Culture conditions were as in Bozdech et al, 2003. Post-synchronization, maximum invasion (number of schizonts = number of rings) was observed by smear and 50mL of 2% hematocrit, 10% parasitemia culture was harvested 44 hours post-invasion (late schizogeny). Harvested cells were centrifuged at 1,500 g for 5 min, washed in phosphate-buffered saline (PBS), and pelleted at 1,500 g for 5 min. The cell pellet was rapidly frozen in liquid nitrogen and stored at −80°C. Total RNA was harvested from the frozen pellet using 10mL Trizol (Invitrogen Corp., Carlsbad, CA). 238ug of total RNA was poly-A selected using the Micro Fasttrack 2.0 kit (Invitrogen Corp., Carlsbad, CA).

#### DNase treatment, reverse transcription, PCR, and sequencing

3.6ug of poly-A selected RNA was treated twice with 2uL of TURBO DNase according to the manufacturer's instructions for the TURBO DNase-free kit (Applied Biosystems/Ambion, Austin, TX). Treated RNA tested negative for residual genomic DNA by PCR amplification in the following mix: 1× Herculase II Fusion buffer, 0.25mM dATP, 0.25mM dTTP, 0.0625mM dCTP, 0.0625mM dGTP, 0.25uM PF11_0062-F primer (5′-ACTGGTCCAGATGGAAAGA AAAA-3′), 0.25uM PF11_0062-R (5′-GGAGGTAAATTTTGTTACAGCTTTGGTTCC-3′), and 0.4uL of Herculase II Fusion polymerase (Stratagene, La Jolla, CA). PCR conditions were 95°C for 2 min, then 40 cycles of 95°C for 30 sec, 52°C for 45 sec, 65°C for 3 min, and finally 65°C for 7 min. 2ug of DNased RNA was melted at 65°C for 5 minutes in the presence of 817.5ng random hexamer, and then cooled at room temperature for 5 minutes. To reverse transcribe cDNA, 0.25mM dATP, 0.25mM dTTP, 0.0625mM dCTP, 0.0625mM dGTP, 1× First Strand buffer, 10M DDT, and 1090U Superscript III reverse transcriptase (Invitrogen, Carlsbad, CA) were added and the reaction was incubated at 42°C for 1.5 hours. 1uL of this reverse transcription mix was used for each junction confirming PCR using the previous described mix and cycling conditions, with the following changes: 0.5uM of the appropriate forward and reverse primers were used and 30 cycles of PCR were performed. Primer sequences are listed in Table 4. PCR reactions were cleaned up with Zymo-5 DNA columns (Zymo Research Corp., Orange, CA). 100ng of each PCR product was a-extended by incubation at 37°C for 30 minutes in the presence of 16.7mM dATP, 1× NEB buffer 2, and 5U Klenow exo^−^ (New England Biolabs, Ipswich, MA). Extended products were then TOPO TA cloned and transformed into chemically competent TOP10 cells (Invitrogen, Carlsbad, CA). Transformations were plated on LB+ampicillin plates spread with 100uL 40mg/mL Xgal. After 16 hours of growth, colony PCR was performed on white colonies with the following PCR mix: 1× Taq buffer, 2mM MgCl_2_, 0.5uM M13F, 0.5uM M13R, 0.25mM dATP, 0.25mM dTTP, 0.0625mM dCTP, 0.0625mM dGTP, and 0.5U Taq polymerase (Invitrogen, Carlsbad, CA). PCR conditions were 95°C for 2 min, then 30 cycles of 95°C for 30 sec, 52°C for 45 sec, 65°C for 3 min, and finally 65°C for 7 min. Following precipitation with 3 volumes of isopropanol, 1/4 of each PCR product was primer extended in Sanger sequencing reactions in the presence of 1uM M13F, 1× sequencing buffer, and 0.5uL BigDye Terminator (Applied Biosystems Inc., Foster City, CA). Cycling conditions were 94°C for 2 min, then 60 cycles of 94°C for 30 sec, 50°C for 1 min, 60°C for 1 min, and finally 60°C for 7 min. Sequencing reactions were precipitated with 1/4 volume 125mM EDTA and 1 volume 100% ethanol, and then resuspended in HiDi formamide and run on a 3130xl Genetic Analyzer (Applied Biosystems Inc., Foster City, CA).

**Table 4:** Primer Sequences.

Name	Sequence
PF07_0101 F	TGGGTTATCTGATCATCAAGGA
PF07_0101 R	TTTTATGAGTGTCGTCCCTTTTT
PFD0185c F1	CGCACTACCATATTTATGCCTCT
PFD0185c R1	AGTAGAAGGAGGGAGGAGCA
PFD0185c F2	TTCGCGTGATGAAGAAGATG
PFD0185c R2	CAAGCCCACATATAAATCAAGGA
PFC0285c F	TATCTTCTTGGGCCCCTTCT
PFC0285c R	TGTGAATGCGTGAAGGATTT

Primers used for experimental validation.
